# Materials and Processes for Schottky Contacts on Silicon Carbide

**DOI:** 10.3390/ma15010298

**Published:** 2021-12-31

**Authors:** Marilena Vivona, Filippo Giannazzo, Fabrizio Roccaforte

**Affiliations:** Consiglio Nazionale delle Ricerche, Istituto per la Microelettronica e Microsistemi (CNR-IMM), Strada VIII, n. 5—Zona Industriale, I-95121 Catania, Italy; filippo.giannazzo@imm.cnr.it (F.G.); fabrizio.roccaforte@imm.cnr.it (F.R.)

**Keywords:** silicon carbide, 4H-SiC, Schottky barrier, Schottky diodes, electrical characterization

## Abstract

Silicon carbide (4H-SiC) Schottky diodes have reached a mature level of technology and are today essential elements in many applications of power electronics. In this context, the study of Schottky barriers on 4H-SiC is of primary importance, since a deeper understanding of the metal/4H-SiC interface is the prerequisite to improving the electrical properties of these devices. To this aim, over the last three decades, many efforts have been devoted to developing the technology for 4H-SiC-based Schottky diodes. In this review paper, after a brief introduction to the fundamental properties and electrical characterization of metal/4H-SiC Schottky barriers, an overview of the best-established materials and processing for the fabrication of Schottky contacts to 4H-SiC is given. Afterwards, besides the consolidated approaches, a variety of nonconventional methods proposed in literature to control the Schottky barrier properties for specific applications is presented. Besides the possibility of gaining insight into the physical characteristics of the Schottky contact, this subject is of particular interest for the device makers, in order to develop a new class of Schottky diodes with superior characteristics.

## 1. Introduction

Nowadays, the wide bandgap semiconductors SiC and GaN are considered as the basis of a huge advancement in power electronics, enabling the definition of a game-changing generation of devices with superior performance if compared with that currently achieved by traditional Si-based devices [1]. This is due to the outstanding physical properties of this class of materials, such as wide bandgap, high critical electrical field and high saturation velocity, that push forward the limits reached by Si-based power electronics [2]. In addition to a more efficient performance, the superior properties of wide-band gap semiconductors also translate into devices able to operate in high temperature, high power and high frequency regimes, with the possibility of extending the field of applications for power electronics [3].

Among the wide bandgap semiconductors, one of the hexagonal polytypes of silicon carbide, i.e., 4H-SiC, plays a pivotal role in power electronics, owing to the excellent quality achieved from the commercially available substrates and epilayers and the high level of compatibility with the existing Si-based device manufacturing facilities and technology. Quantitatively, 4H-SiC features a wide bandgap of 3.26 eV, high critical electric field >2 MV/cm, high thermal conductivity of 4.9 WK^−1^cm^−1^ and saturated drift velocity higher than 2 × 10^7^ cm s^−1^ [4], making it the material of choice for a variety of power devices operating in the medium/high voltage range (600–3000 V) [3].

Along the lines of Si-based technology, several 4H-SiC power devices have been developed, with a mature technology level in terms of processing implementation and real-world applications [5]. Among them, n-type 4H-SiC-based Schottky barrier diodes (SBDs) are currently commercialized on a large scale in the power electronics market and have penetrated our daily lives in various fields, e.g., consumer electronics, electric and hybrid vehicles, industrial processes, energy conversion from renewable sources, sensors, photodetectors and so on [6,7,8].

**Figure 1:**
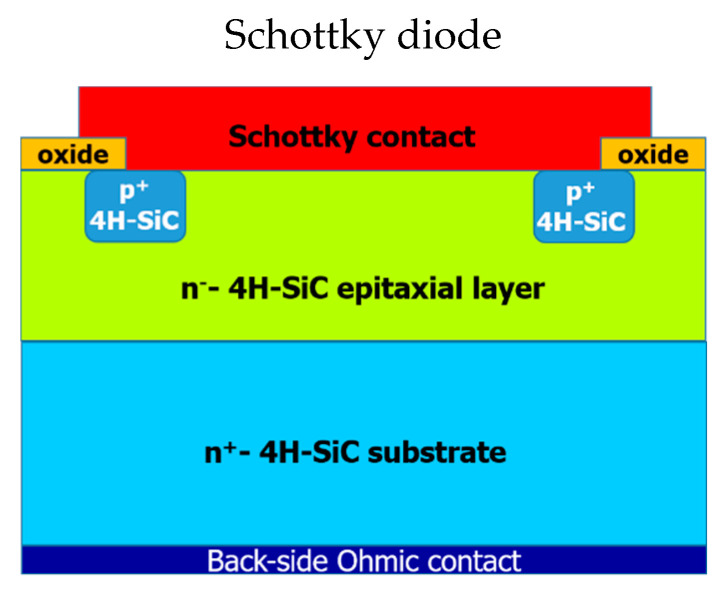
Schematic cross-section view of a 4H-SiC Schottky barrier diode (SBD).

A schematic cross-section view of a typical SBD is depicted in Figure 1. In this device, the drift layer is given by a lightly doped n-type 4H-SiC epitaxial layer (doping density *N_D_* ~ 10^16^ cm^−3^) grown onto a heavily doped n-type 4H-SiC substrate (*N_D_* > 5 × 10^18^ cm^−3^). The connection with external components is given by metal electrodes consisting of a front-side Schottky contact to the 4H-SiC epitaxial layer and a back-side Ohmic contact to the 4H-SiC substrate. In addition, the Figure schematically illustrates a p-type implanted edge termination needed to optimize the breakdown behavior. For the sake of completeness (not reported in the scheme), a few μm thick front-side metallization (typically Al based) acting as device bonding pad and a polymeric passivation finalize the top part of the device, while a thick metal layer, soldered to the back-side and consisting of a gold-plated metal frame wrapped in ceramic, operates as connection to the package. A schematic fabrication process flow, with the sequential steps typically adopted for the Schottky diode fabrication, can be found in [9].

The core of an SBD is the metal/semiconductor junction (the so-called Schottky contact) and the properties of this system must be carefully investigated to assess and optimize the electrical performance of the whole device [9].

Over the years, different approaches, ranging from the choice of materials for the Schottky barrier formation to semiconductor treatments or even considering the device layout, have been developed to improve and gain control on the Schottky contact properties. Moreover, unconventional methods were also explored as alternative solutions in improving and controlling the Schottky barrier systems.

In this paper, after a brief discussion on the fundamentals of the metal/4H-SiC Schottky barrier formation and its electrical characterization, we will give an overview on the current materials and processing solutions for the fabrication of Schottky contacts to 4H-SiC. Afterwards, besides the consolidated approaches, we will present a variety of the nonconventional methods proposed in literature to control the Schottky barrier properties for specific applications.

## 2. Schottky Contacts to n-Type 4H-SiC

In the last three decades, many efforts have been devoted to the study and characterization of the metal/4H-SiC interfaces. Despite the large amount of literature data published on this system, the physics at the base of the Schottky properties and carrier transport through the contact is not yet fully understood, and the research on Schottky contacts to 4H-SiC remains a scientifically open topic. Besides its fundamental scientific character, this research is also strongly pushed by industrial requirements for achieving a more efficient performance of SBDs. In this Section, we briefly report on the Schottky barrier fundamentals, the well-established and the most promising metallization schemes, discussing also some aspects related to the device layouts proposed to optimize the electrical properties of the diodes.

### 2.1. Fundamentals on Schottky Barriers

When metal and semiconductor are put in intimate contact, a Schottky barrier forms, whose height (*ϕ_B_*) is the most significant electrical parameter associated to this system. According to the well-known Schottky–Mott rule [10,11], the Schottky barrier height ideally depends only on the metal work function (*W_M_*) and semiconductor electron affinity (*χ_S_*) [12], as expressed by:

(1) $$\phi_{B}=W_{M}-\chi_{S}.$$

Hence, considering the work function of the typical metals used for SBDs (in the range 4–5.5 eV [13]) and the electron affinity of 4H-SiC (*χ_SiC_* = 3.2 eV [14]), Schottky barrier height values between 1.0 and 2.3 eV are expected, i.e., much higher than in silicon. However, in real cases, the Schottky barrier height does not simply obey to the Schottky–Mott rule, but may depend on other factors, like the surface preparation, metal deposition techniques and/or post-metallization annealing treatments [15,16]. In particular, the electronic properties of the metal/semiconductor contact are affected by the presence of surface states, which can be related to roughness, surface contaminants, residual thin interfacial oxide layers and so on, and are responsible for a deviation from the Schottky–Mott prediction [17,18].

The experimentally measured value of the Schottky barrier height *ϕ_B_* also depends on the methods used for its determination [19]. Generally, the barrier height in a metal/semiconductor system can be determined by means of different techniques, such as electrical characterizations (current–voltage (I–V) and capacitance–voltage (C–V) measurements) or internal photoemission (IPE) measurements that are based on the photo-generated current detection [19,20]. Obviously, each method shows advantages and drawbacks. The electrical characterization methods require the fabrication of appropriate test patterns, namely Schottky diodes, and provide interesting insights of the Schottky barrier nature. For example, the I–V characterization is very sensitive to the presence of inhomogeneity of the barrier, with some well-established models, such as the Tung’s model [17]) or the Werner’s and Güttler’s model [21], developed to take this aspect into account. On the other hand, the C–V characterization supplies information about the space-charge region width [22]. As a consequence, for a given Schottky contact, the barrier height value extrapolated by I–V analysis is typically lower than that derived from the C–V characteristics. This aspect can be explained by the fact that lower barrier height regions are preferential paths for the current, while C–V characteristics account for an overall electrical behavior with the largest regions (usually with highest barrier) dominating in the barrier extraction [22]. On the other hand, IPE measurements are independent of the local barrier inhomogeneity and supply a reliable value for the Schottky barrier. However, the photocurrent detection requires special equipment and semi-transparent front or back contacts, thus making these kind of measurements less common with respect to the electrical characterizations.

The Schottky barrier height determines the electrical behavior of a metal/semiconductor contact by governing the current flow through the metal/semiconductor interface. Generally, for doping density *N_D_* in the range 1 × 10^15^ < *N_D_* < 1 × 10^17^ cm^−3^, under forward voltage the current transport mechanism through the metal/4H-SiC interface is ruled by the thermionic emission (TE) theory. In this model the current *I_TE_* can be expressed by [12]

(2) $$I_{TE}=AA^{*}T^{2}\exp\left[\left(-\frac{q\phi_{B}}{k_{B}T}\right)\right]\exp\left[\left(\frac{qV_{F}}{nk_{B}T}\right)-1\right]$$

where *A* is the device area, A* is the effective Richardson’s constant (146 A·cm^−2^·K^−2^ for 4H-SiC [23]), *k_B_* is the Boltzmann’s constant, *q* is the elementary charge, *V_F_* is the applied forward voltage and *T* is the absolute temperature.

As can be seen in Equation (2), besides the Schottky barrier height *ϕ_B_*, another important electrical parameter that characterizes the Schottky contact is the ideality factor *n*. These parameters can be determined by the intercept and the slope of a linear fit in semilog scale of the forward current–voltage characteristic, using Equation (2) for *V_F_* > 3 *k_B_T*/*q*, where the term −1 can be neglected.

Basically, the TE theory assumes a temperature-independent ideality factor and barrier height. However, in order to justify the experimentally observed temperature dependence of these parameters in real 4H-SiC Schottky contacts, the TE theory was modified, introducing models taking into account local fluctuations (inhomogeneity) of the Schottky barrier over the contact interface, as discussed in the Tung’s [17] model or in the Werner’s and Güttler’s [21] model. Specifically, the Tung’s model [17] assumes a local lateral inhomogeneity at nanometric scale by considering the presence of low barrier regions (patches) embedded in a high-barrier background, while the Werner’s and Güttler’s [21] model considers a Gaussian distribution of barrier heights around an apparent temperature-dependent barrier height.

On the other hand, when the doping concentration of the semiconductor exceeds 10^17^ cm^−3^, the high electric field at the interface and the thin barrier width make dominant for the current transport a thermionic filed emission (*TFE*) mechanism, which involves a tunneling component for thermally excited electrons [19,24]. Specifically, for doping in the range 1 × 10^17^ < *N_D_* < 10^19^ cm^−3^, the *TFE* describes the electrical behavior of the system [24], according to the following current–voltage relationship [25]:

(3) $$I_{TFE}=I_{0,TFE}\left(V_{F}\right)\times \exp\left(q\frac{V_{F}}{E_{0}}\right)$$

The term *I*_0,*TFE*_ *(V_F_)* corresponds to the saturation current and is given by

(4) $$I_{0,TFE}\left(V_{F}\right)=\frac{AA^{*}T}{k_{B}cosh\left(qE_{00}/k_{B}T\right)}\times \sqrt{\pi E_{00}\left(\phi_{B}-\Delta E_{F}-V_{F}\right)}\times \exp\left(-\frac{q\Delta E_{F}}{k_{B}T}-\frac{\phi_{B}-\Delta E_{F}}{E_{0}}\right)$$

with $E_{0}=E_{00}\times coth\left(\frac{qE_{00}}{k_{B}T}\right)$ dependent on the doping concentration *N_D_* through the parameter $E_{00}=\frac{h}{4\pi }\times \sqrt{\frac{N_{D}}{m^{*}\varepsilon_{SiC}}}$. The other symbols are the Planck’s constant *h*, the effective mass m* = 0.38 *m*_0_ (with m_0_ the electron mass) [26] and the dielectric constant of the semiconductor *ε_SiC_* = 9.76 *ε*_0_ (with *ε*_0_ the vacuum permittivity) [4]. Δ*E_F_* is the difference between the bottom of the conduction band and the semiconductor Fermi level. In this case, the barrier *ϕ_B_* and the doping concentration *N_D_* can be considered the parameters to be determined from a best-fit procedure of the experimental detected current–voltage characteristics.

**Figure 2:**
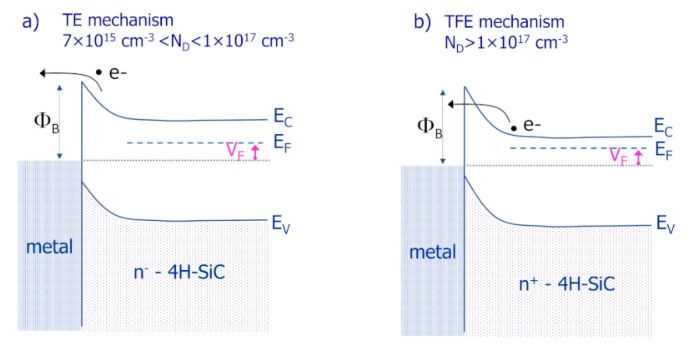
Schematic energy band diagrams for the metal/4H-SiC contact under forward bias *V_F_*, according to the predominance of the (**a**) thermionic emission (TE) or (**b**) thermionic field emission (*TFE*) current transport mechanism.

Figure 2 depicts a schematic energy band diagram for a metal/semiconductor junction under forward bias *V_F_*, when the predominant current transport mechanism is ruled by the TE (Figure 2a) or by the *TFE* (Figure 2b) model, according to the doping of the semiconductor layer at the interface. Noteworthy, as shown in Equations (2)–(4), in both models, the current has an exponential dependence on the Schottky barrier height. Thus, a good control of the *ϕ_B_* must be guaranteed, as it significantly affects the current level through the contact.

### 2.2. Survey of Literature Data on Schottky Contacts to n-Type 4H-SiC

In literature, many studies on the metal/n-type 4H-SiC systems have been reported and focused on the choice of the metal and its evolution in the Schottky contact formation [27,28,29,30,31,32,33]. A collection of literature results related to some of the most diffused metal/n-type 4H-SiC contacts is reported in Table 1, including as deposited (unannealed) Schottky contacts or contacts subjected to thermal annealing treatments. The reported barrier height values were determined by I–V measurements on Schottky diodes.

**Figure 3:**
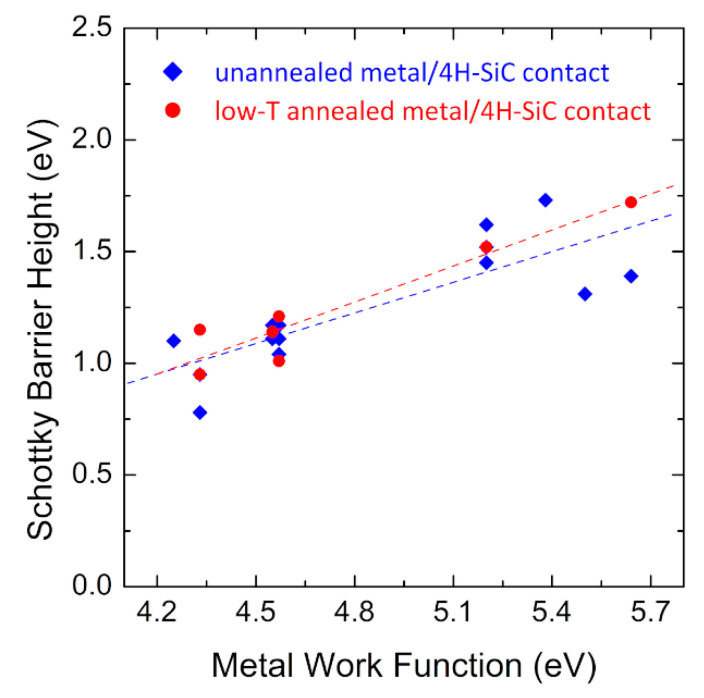
Experimental dependence of the barrier height *ϕ_B_* on the metal work function *W_M_* in unannealed and low-temperature annealed metal/n-type 4H-SiC systems. All the reported barrier values were determined by I–V characterization of Schottky diodes. Data are taken from Refs. [21,22,23,24,25,26,27,28,29,30,31,32,33,34,35,36,37,38,39,40,41,42,43,44].

As can be seen, a large variety of barrier height values is found, depending on the metals and post-metallization thermal treatments. Especially, by reporting the barrier height values *ϕ_B_* versus the metal work function *W_M_*, it is possible to determine the correlation between *ϕ_B_* and *W_M_*, which represents the so-called “interface index” S = d*ϕ_B_*/d*ϕ*_m_ [45]. Typically, for real 4H-SiC-based Schottky contacts, a linear correlation is found, with S values between the Bardeen limit (i.e., S = 0, indicating interface properties independent of the metal) and the ideal Schottky–Mott behavior (S = 1) [4,46], suggesting the occurrence of a partial Fermi level pinning at the interface. Figure 3 displays this kind of plot for the unannealed and low-temperature annealed metal/4H-SiC contacts, with the values taken from Table 1. The slope values confirm an intermediate behavior, with a slight improvement towards the Schottky–Mott behavior from 0.46 to 0.54 for the low-temperature annealed contacts.

**Figure 4:**
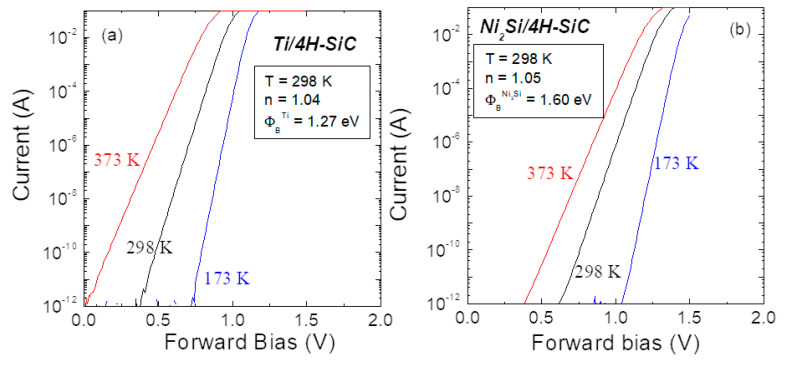
Semilog plot of the temperature-dependent forward-voltage characteristics of 4H-SiC Schottky diodes based on (**a**) Ti and (**b**) Ni_2_Si. Adapted with permission from Ref. [23]. Copyright 2021 AIP Publishing.

Throughout the years, titanium (Ti) and nickel silicide (Ni_2_Si) have come out as widely diffused barrier metals for 4H-SiC Schottky diodes in different applications. Ti- and Ni_2_Si-based metallization schemes are currently well-established technology, offering a high level of reproducibility for the Schottky barrier height values. The representative forward I–V characteristics of the 4H-SiC Schottky diodes, employing Ti and Ni_2_Si barrier metals and acquired at three different temperatures (173, 298 and 373 K), are reported in Figure 4a,b, respectively [23,47]. All the curves were analyzed according to the TE model, by fitting the linear region in a semilog plot of the forward I–V curve according to Equation (2) approximated for the linear region. From this analysis, the extrapolation of an ideality factor *n* very close to 1 for both contacts confirms the predominance of the TE mechanism in the current transport through the metal/semiconductor interface. Specifically, the values of the Schottky barrier height typically obtained at room temperature were *ϕ_B_* = 1.27 eV for the Ti/4H-SiC and 1.60 eV for the Ni_2_Si/4H-SiC contacts [23,47]. Consequently, Ti is used as the Schottky barrier material in power electronics applications, for which a low barrier height is desired [48], whereas Ni_2_Si is preferred for sensing or detection applications [49,50], where the higher barrier permits a low leakage current to be obtained. Moreover, the possibility of a “self-aligned” process given by the Ni_2_Si formation is used for the fabrication of semi-transparent interdigit electrodes in UV-detectors [51].

### 2.3. Diode Layout

Managing the power dissipation in Schottky diodes is becoming an aspect of increasing interest, due to the need to reduce electricity consumption in modern electronic systems. In particular, a conventional Schottky diode (as that schematically depicted in Figure 1) is a majority carrier device, in which the dynamic power dissipation is negligible with respect to the conduction static power losses [52]. In such a device, the barrier height, ideality factor and reverse leakage current are important parameters affecting the static power dissipation. In particular, the conduction static power dissipation *P_D_* can be expressed as [53]:

*P_D_* = %*_ON_* × (*V_F_* × *J_F_*) + (1 − %*_ON_*) × (*V_R_* × *J_R_*) (5)

where *%_ON_* is the ON duty cycle, *V_F_* and *J_F_* are the voltage and current density under forward bias, while *V_R_* and *J_R_* are the voltage and current density under reverse bias. Hence, considering the case of the conventional 4H-SiC Schottky diodes, the forward current-voltage behavior is described by the TE model, with the *V_F_* expressed as function of the forward current density *J_TE_* as follows, accordingly to the linear region approximation of Equation (2):

(6) $$V_{F,TE}\left(J_{TE}\right)=n\phi_{B}+\frac{nkT}{q}ln\left(\frac{J_{F}}{A^{*}T^{2}}\right)$$

Moreover, due to the high electric field in the space-charge region that entails a sharp band bending and thus a thin barrier, the reverse current is typically described according to the *TFE* model, with the relationship between current density *J_TFE_* and reverse voltage *V_R_* given by

(7) $$J_{R,TFE}\left(V_{R}\right)==A^{*}T^{2}\sqrt{\frac{q^{2}\pi E_{00}}{{\left(kT\right)}^{2}}}\sqrt{V_{R}+\frac{\phi_{B}}{cosh^{2}\left(\frac{qE_{00}}{kT}\right)}}\;\exp\left(-\frac{\phi_{B}}{E_{0}}\right)\exp\left(\frac{V_{R}}{E_{1}}\right)$$

where $E_{1}=E_{00}\times {\left(qE_{00}/kT-tanh\left(qE_{00}/kT\right)\right)}^{-1}$ and the other parameters of Equation (7) as described before. Hence, accordingly to Equation (5), the static power dissipation depends on the Schottky barrier height *ϕ_B_*.

**Figure 5:**
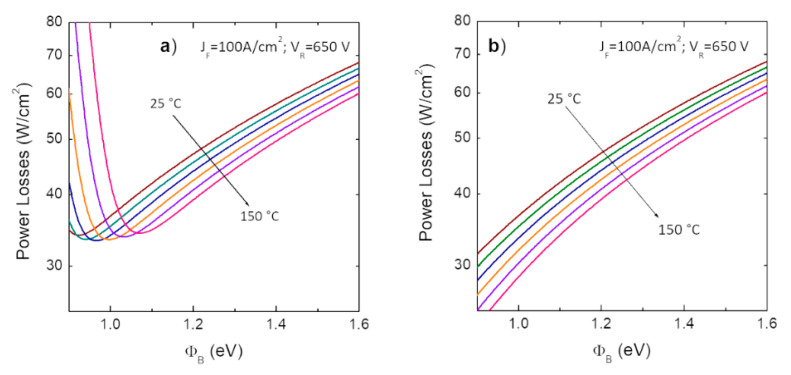
Schottky barrier height dependence of the static power losses for (**a**) a conventional Schottky diode and (**b**) a modern JBS diode, in the temperature range 25–150 °C. The curves were simulated by considering the forward electrical behavior ruled by thermionic emission model and the reverse characteristics ruled by thermionic field emission model. In the case of the JBS diode (**b**), the leakage current contribution has been neglected.

Figure 5a shows the calculated conduction power loss for conventional 4H-SiC Schottky diode as a function of the barrier height, in the temperature range 25–150 °C. The calculation has been performed using Equations (5)–(7), assuming a duty cycle %ON = 50%, a forward current density *J_F_* = 100 A/cm^2^ and reverse voltage *V_R_* = 650 V. As highlighted in Figure 5a, for a given temperature, a reduction of the Schottky barrier entails a reduction of the power losses, up to a certain lower limit, where the losses show a sudden increase, due to the significant increase of the reverse leakage current with the barrier reduction, especially at higher temperatures. Evidently, based on these considerations, in power electronics applications, Schottky contacts with low barrier height are sought after, as lowering the Schottky barrier height leads to a reduction of the power consumption [48]. However, it must be considered that a lowering of the barrier *ϕ_B_* could lead to an increase of the leakage current density *J_R_* and thus, a good compromise between the diode forward and reverse behavior must be found for the minimization of the power dissipation *P_D_*.

For that reason, as discussed later in this paragraph, in modern 4H-SiC-device technology, the high-reverse *TFE* leakage current, which typically characterized the conventional Schottky diodes, has been strongly reduced by acting on the device layout. In this case, the contribution of the leakage current can be neglected, and the static conduction losses decrease with a reduction of the barrier height, as shown in Figure 5b.

**Figure 6:**
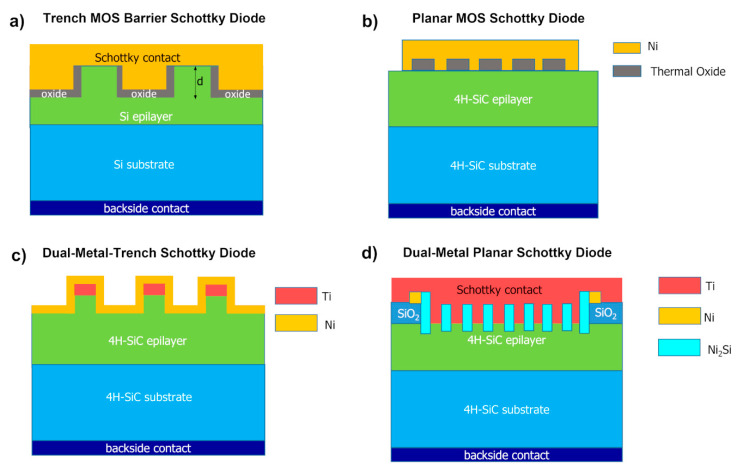
Different Schottky diode layouts proposed to achieve an optimal trade-off between the forward and reverse characteristics. (**a**) Trench MOS barrier Schottky (TMBS) diode. (**b**) Planar MOS Schottky diode (MOSSD). (**c**) Dual-metal-trench (DMT) Schottky diode with Ti and Ni Schottky contact. (**d**) Dual-metal-planar (DMP) Ti-Ni_2_Si/4H-SiC Schottky diode.

Since the middle of the 1990s, new diode designs have been proposed to achieve improved rectifier characteristics of semiconductor-based Schottky diodes. Mehrotra et al. [54] demonstrated that a design involving metal-oxide-semiconductor (MOS) regions, built into a trench region of the device front, was successful in pushing higher the limit given by the reverse blocking voltage, allowing the device to support larger doping of the semiconductor epitaxial layer, and thus an improvement of the on-state characteristics. A schematic view of this device is reported in Figure 6a: as can be seen, the MOS structure is formed on the bottom and sidewalls of a trench, while the Schottky contact is on the top surface. With this layout, called trench MOS barrier Schottky rectifier (TMBS), a reduced level of electrical field is achieved on the Schottky interface, producing a smaller Schottky barrier height lowering and thus a reduced leakage current level, if compared with the standard Schottky structure.

Afterward, another approach of “Schottky-pinch rectifier” was proposed by Zhang et al. [55] for 4H-SiC Schottky rectifiers. Differently from the previously discussed solution, this diode layout consisted of integrating MOS-structures (with a thermally grown oxide) together with Ni Schottky contacts on the same plane, as shown in Figure 6b. This layout, called the planar MOS Schottky diode structure (MOSSD), was able to maintain an acceptable level of forward current, up to 90% with respect to a conventional Schottky diode of the same footprint area, while reducing the leakage current by one order of magnitude.

However, these designs including MOS structures, could give some limitations, with the possible occurrence of the oxide breakdown before the 4H-SiC critical electrical field was reached. To overcome this limitation, a dual-metal-trench (DMT) device structure, implementing low and high Schottky barrier height materials (i.e., Ti and Ni metals, respectively) was suggested by Schoen et al. [56]. The device scheme is given in Figure 6c: under forward bias, the mesa was not pinched off and the electrical characteristics are given by the low barrier contact (Ti/4H-SiC). In contrast, under reverse bias, the mesa structure became fully pinched-off and the high barrier height of the Ni Schottky contact prevails, limiting the electric field. A further evolution of the DMT has been later proposed by Roccaforte et al. [57], who combined the advantages of Ni_2_Si and Ti in a dual-metal-planar (DMP) Schottky diode (schematically depicted in Figure 6d) which exhibited a forward voltage drop close to that of a Ti/4H-SiC diode (lower barrier) and a reverse current comparable to that of a Ni_2_Si/4H-SiC (higher barrier).

The electrical behavior of the DMP structure can be explained by an equivalent system with two parallel diodes which have two different barrier heights, specifically the low barrier of the Ti layer determines the current flow under forward bias, and the high Ni_2_Si barrier dominates the reverse conduction by the pinch-off of the low barrier Ti regions. The DMP diode presented an ideality factor *n* = 1.25, a barrier height *ϕ_B_* = 1.23 eV (close to that of Ti contact) and leakage current at −100 V of 5.8 × 10^−4^ A/cm^2^, 30 times lower than the leakage current observed in the Ti/4H-SiC diode.

All the aforementioned diode layouts showed an improvement of the trade-off between the forward and reverse characteristics of the diodes.

**Figure 7:**
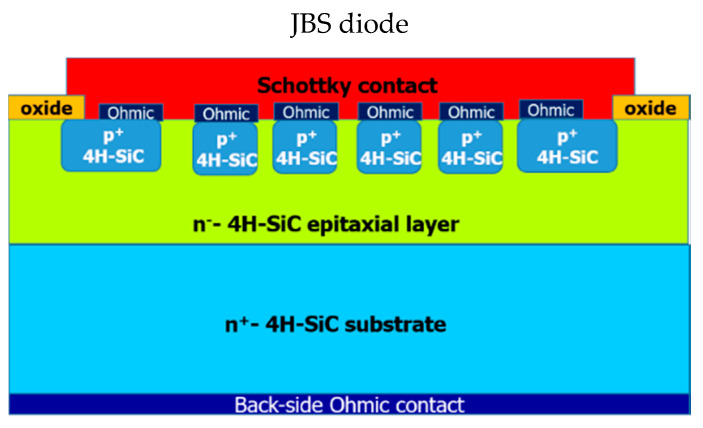
Schematic cross-section view of a 4H-SiC junction barrier Schottky (JBS) diode.

Nowadays, the so-called junction barrier Schottky (JBS) diode is the most widely used Schottky-like architecture in SiC technology with significant improvement with respect to the standard Schottky diode [58]. This device consists in p^+^-type regions (usually achieved by p-type ion implantation and electrical activation) embedded within an n-type Schottky epitaxial area, as schematically shown in Figure 7 [59,60]. This layout mitigates the reverse leakage current of the Schottky diode and achieves a hard breakdown, as typical of a p-n junction [58]. Specifically, under low forward bias, the current flows in the regions between the p^+^-wells, exploiting the Schottky barrier characteristics given by the top metal, while under reverse bias, these regions are pinched-off and the electrical characteristics are given by the p-n junction. The distance *d* between two adjacent p^+^-wells and the size *s* of p^+^-well are important parameters that must be carefully designed for optimizing the trade-off between forward and reverse characteristics. These parameters, together with the depletion width *W_D_*, defined the cell pitch *p* (*p = d + s + W_D_*). For instance, the on-state voltage drop decreases as the cell pitch is reduced, while the leakage current decreases as the p^+^-well distance is reduced for a constant value of p^+^-well size [61]. The schematic process flow, with the sequential steps typically adopted for JBS diode fabrication, can be found in Ref. [9]. As occurs in SBDs, in these devices the Schottky contact between the metal and the n-type epitaxial 4H-SiC is also a key part for optimizing the overall electrical performance of the device.

Beyond the diode layout with the well-established JBS design for 4H-SiC-based Schottky rectifiers, the employment of materials with low work function is of particular interest for minimizing the power dissipation of Schottky diodes and they are currently explored with promising results. This aspect will be discussed in detail in the next subsection.

### 2.4. Low Work Function and Refractory Metals

In the last years, regarding the choice of the Schottky metal for 4H-SiC-JBS, increasing interest has been devoted to metallization schemes containing metals (and their compounds) with low work function and a certain degree of stability with 4H-SiC and the environment. In the facts, these low work function materials (such as the refractory metals Mo, W, Nb, etc.) can guarantee a minimization of the on-state conduction losses, making this configuration highly aimed at industrial 4H-SiC-based Schottky device development [48]. Moreover, since these metals exhibit a high melting point, they could be indicated for harsh environment applications, requiring temperature-resistant materials [62].

In recent literature, many papers have dealt with the electrical characterization of Mo/4H-SiC Schottky contacts for power electronics [63,64,65,66,67], highlighting the possibility of achieving a barrier height value as low as 1.010 eV and an ideality factor of 1.045 [65]. As reported in those studies, the Mo/4H-SiC behaved as an inhomogeneous contact, with the current conduction dominated by a TE mechanism and a slight discrepancy from the ideal behavior explained either according to the Werner and Güttler [21] or the Tung model [17].

**Figure 8:**
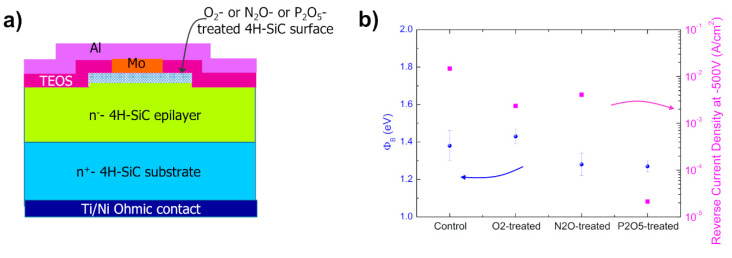
(**a**) Schematic view of Mo/4H-SiC Schottky diodes with premetallization treatments of the semiconductor surface, consisting of thermally grown oxidation in O_2_ and N_2_O or oxide deposition of P_2_O_5_, followed by oxide removal prior to Mo deposition in all cases. (**b**) Schottky barrier height and reverse leakage current density at −500V values averaged over a set of *I*–*V_F_* curves of equivalent Mo/4H-SiC Schottky diodes with 4H-SiC surface pretreated under different conditions. Panel (**b**) is adapted with permission from Ref. [63]). Copyright 2021 AIP Publishing.

Very recently, Renz et al. [63] studied a series of surface passivation treatments to achieve an improvement of the Mo/4H-SiC Schottky diode electrical properties. In particular, after the deposition or thermal growth of an oxide layer, annealing processes, similar to those employed in metal-oxide-semiconductor field-effect transistors (4H-SiC MOSFETs) technology, were considered [68,69,70]. These treatments included thermal oxidation in O_2_ or N_2_O environments at temperatures of 1400 and 1300 °C, respectively, or the deposition of a phosphorus pentoxide (P_2_O_5_) layer at 1000 °C for 2 h. The first two processes consume the SiC surface while the third one does not. In all the samples, the oxide on the surface was removed by cleaning in dilute HF (10%) solution prior to Mo Schottky metal deposition. Figure 8a shows this approach schematically, with the treated area of the semiconductor depicted as a patterned blue layer. An electrical *I*–*V_F_* characterization at room temperature was performed on a set of equivalent Mo/4H-SiC diodes fabricated under different conditions and, for comparison, on an untreated Mo/4H-SiC contact (labelled as “control”). The *I*–*V_F_* curves were analyzed according to TE model, obtaining an almost ideal behavior after the treatments. The lowest barrier height value (*ϕ_B_* = 1.27 ± 0.03 eV) was observed for the contact subjected to a prior deposition of P_2_O_5_ (left scale of Figure 8b). Surprisingly, although a reduced value of barrier height was obtained in the P_2_O_5_-treated contacts with respect to the control sample, this process enabled the lowest value of the leakage current (right scale of Figure 8b) to be obtained. The authors explained this effect with the capability of the oxide to homogenize the interface by filling the nanopits, as witnessed by means of morphological (AFM) and microstructural (TEM) analyses. The high density (5 × 10^9^ cm^−2^) of these nanopits allows one to believe that they are different from those typically related to the threading dislocations arriving on the surface of 4H-SiC and observed after removal of surface electrodes [71]: if the threading dislocation-related nanopits were demonstrated to be potential leakage paths for the current, plausibly, in the case of treated P_2_O_5_ deposited on 4H-SiC surface, the nanopits were oxide-filled, with a barrier lowering due to two contributions, one associated with the phosphorous-rich region below the contact, which increases the n-type doping, and the other related to a homogenization of the barrier height after oxide termination of the surface defects. This could explain the reduction of the barrier, with a simultaneous decreasing of the leakage current. It is worth noting that the absence of silicide reaction at the Mo/SiC interface, which would otherwise consume the top few nanometers of 4H-SiC, enabled these beneficial changes in the contact subsurface.

Furthermore, Mo-based Schottky contacts were also investigated as a possible route for an improved control of the Schottky contact properties. For instance, Stöber et al. [72] proposed the use of molybdenum nitride (MoNx) thin film metallization for adjusting the barrier height within a large range by varying the nitrogen N_2_ fraction in the reactive sputtering metal deposition step in the fabrication process. The total gas flow, i.e., sum of argon and nitrogen, was kept constant at 80 sccm while the nitrogen fraction *χ* = N_2_/(Ar + N_2_) in the gas composition varied from 0 to 80%, by increasing the content of N_2_ in the chamber. For a pure Mo contact (with *χ* = 0%), the Schottky barrier was *ϕ_B_* = 0.68 eV at room-temperature, increasing up to 1.03 eV for *χ* = 80%. Due to the polycrystalline nature of the Mo_2_N thin films, the barriers showed an inhomogeneous behavior, probably arising from different microstructures with regard to the nitrogen fraction used in the processing.

In parallel to Mo-based 4H-SiC Schottky contacts, Schottky contacts based on W have also been largely studied, obtaining a Schottky barrier ranging between 0.94 to 1.29 eV [62,73,74]. According to these papers, a certain degree of inhomogeneity was observed in the W/4H-SiC Schottky contact, successfully explained by means of the Tung’s model [73] or the Werner and Güttler’s model [74].

Noteworthy, for both Mo and W metals, also the carbide compounds were considered as possible electrode material [75,76,77,78]. As an example, Knoll et al. [76] investigated a Schottky barrier based on tungsten carbide, fabricated by depositing a thin layer of W (2 nm) followed by a rapid thermal annealing in vacuum for 5 min at temperatures ranging from 600 to 1200 °C. Then, a 500 nm thick Al layer was deposited on the top of the structure to define the diode structures. At temperatures > 1000 °C, a W_2_C hexagonal structure layer in epitaxial relation with 4H-SiC was produced, stable up to the highest tested temperature of 1200 °C. In this system, they observed a barrier height of 0.94 eV extrapolated under forward I–V characterization.

**Figure 9:**
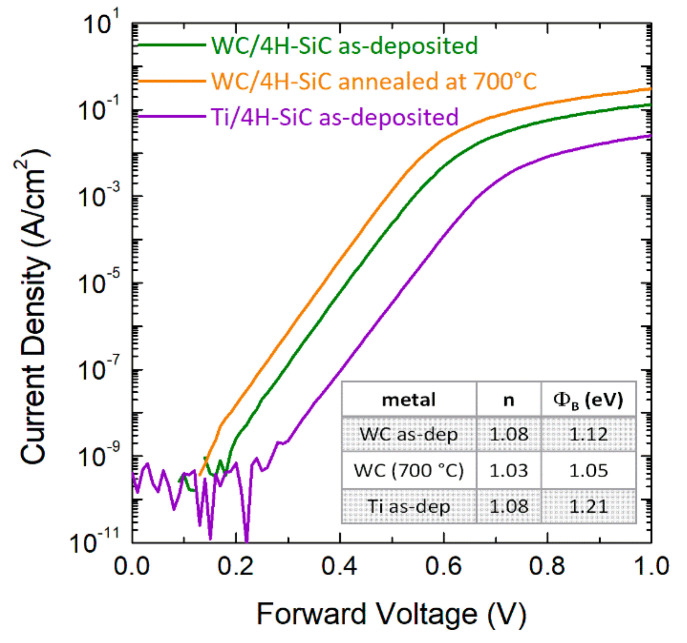
Representative forward I–V characteristics of WC/4H-SiC Schottky diodes for the as-deposited contact and after thermal annealing at 700 °C. The I–V characteristic of a reference Ti/4H-SiC is also reported for comparison. The data are taken from Refs. [78,79].

Recently, we investigated 4H-SiC Schottky diodes with an 80 nm thick layer of tungsten carbide (WC) barrier metal, deposited by magnetron sputtering and defined by optical lithography and lift-off [39,78] process. The Schottky diodes were characterized both before (as-deposited) and after some annealing treatments with temperatures varying form 475 °C to 700 °C for 10 min in N_2_ atmosphere by I–V measurements and applying the thermionic emission (TE) model to the analysis of the electrical characteristics. The Schottky barrier height *ϕ_B_*, derived by fitting the linear region of the semilog forward *J*–*V_F_* curves reported in Figure 9, had an average value of 1.12 eV in the as-deposited contact and decreased down to about 1.05 eV after annealing at 700 °C. In our experimental conditions, the ideality factor was only slightly affected by the annealing treatment, with a value decreasing from 1.08 to 1.03. For sake of comparison, we also reported in the same Figure 9, a representative *J*–*V_F_* curve of a similar Ti/4H-SiC Schottky contact, for which a barrier height of 1.21 eV was observed [79].

In those studies, we observed a temperature-dependence of the *ϕ_B_* and *n*, extrapolated by means of I–V–T characterization, for the annealed-WC/4H-SiC contact [78], as well as for the W/4H-SiC contact fabricated and annealed under similar conditions [39]. This indicated the presence of a nanoscale lateral inhomogeneity for both Schottky contacts, that was fully described by means of the Tung’s model, with an effective barrier *ϕ_Beff_* of 1.15 and 0.96 eV and a homogeneous barrier *ϕ_B_*_0_ of 1.28 and 1.11 eV for the W/4H-SiC and WC/4H-SiC contact, respectively.

Essentially, the promising results obtained for the 4H-SiC Schottky diodes based on these low-work function refractory materials (mainly W and Mo) enable the study of this kind of contacts to be pushed forward towards a better understanding of the Schottky properties and inhomogeneity, and a suggestion of possible solutions for a better barrier uniformity and interface quality.

## 3. Unconventional Approaches for the Control of 4H-SiC Schottky Interfaces

Parallel to the standard metallization stacks and layouts presented in the previous Section, a variety of innovative contacts, chemical compounds or alternative metal stacks have been proposed as new routes to control the Schottky barrier height values on 4H-SiC. In the next subsections, we will discuss some of the representative papers on these unconventional methods.

### 3.1. Manipulation of the Schottky Interface

**Figure 10:**
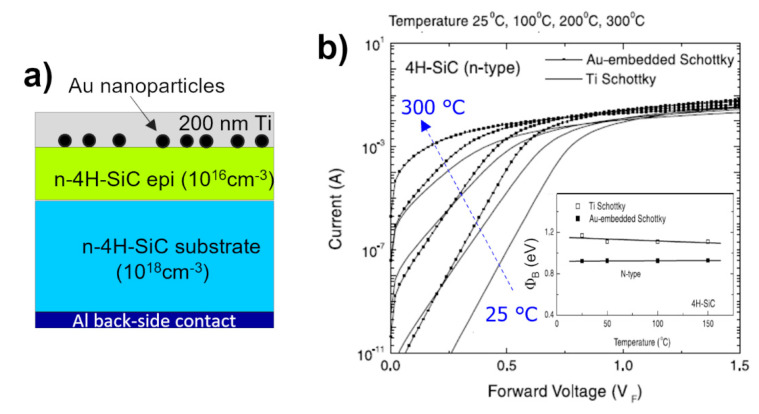
(**a**) Schematic view of Ti/4H-SiC Schottky contact with embedded Au nanoparticles on 4H-SiC. (**b**) Forward current–voltage characteristics of Au-nanoparticle embedded Ti/4H-SiC contact and particle-free Ti/4H-SiC control contact for different measurement temperatures (25, 100, 200 and 300 °C). Inset: comparison between the Schottky barrier height value of Au-nanoparticle-embedded Ti/4H-SiC contact and Ti/4H-SiC control contact, as a function of the measurement temperature. Figures adapted with permission from Ref. [80]. Copyright 2021 Elsevier Ltd.

Since the early 2000s, some studies demonstrated the possibility of lowering the barrier height in the Schottky diode by the incorporation of nanostructures in the metal layer of the Schottky contact [80,81,82,83]. One of the first attempts in SiC was reported by Lee et al. [80], who studied the effect of Au-nanoparticle embedding in Ti/n-type 4H-SiC contact. The diodes were fabricated by first depositing Au-aerosol nanoparticles (diameter of 20 nm, density of 90 μm^−2^) and then depositing 200 nm thick Ti layer in an evaporation chamber. The schematic view of the final Schottky diode structure is depicted Figure 10a. In Figure 10b, the I–V curves for these Schottky-diode embedding nanoparticles are compared to those of a control Ti/4HSiC Schottky diode.

From the comparison, carried out at four different measurement temperatures (25, 100, 200 and 300 °C), it was possible to point out that for each testing temperature, the I–V curve related to the Au-nanoparticle-embedded Ti/4H-SiC contact was shifted towards a lower voltage than the control sample (Figure 10b). The Schottky barrier height, derived by a fit in the linear region according to TE theory and reported in the inset of Figure 10b, was lowered of 0.19 eV in the sample with nanoparticles. To explain this result, the authors invoked the enhancement of the electric field under the interface in the depletion region, due to the small size of the embedded particles and the large Schottky barrier height difference obtained by using two metals as Ti and Au. This is, in part, confirmed by theoretical calculation according to the Tung’s dipole-layer approach [18].

Later on, other studies reported on Schottky contacts with Au- or Ag-nanoparticles embedded in a Ni-metal [82] or Al-metal [83] layer, obtaining a similar reduction of the Schottky barrier height, which was associated with a reduction of the metal work function induced by the presence of the interfacial nanoparticles.

Besides working on the barrier material, many efforts have been dedicated also to the preparation and treatments of the semiconductor surface, to obtain a higher degree of homogeneity of the contact.

As an example, the inhomogeneity observed in Schottky contacts to 4H-SiC could be reduced by suitable treatments of the semiconductor surface, such as passivation with the insertion of an insulating thin film between the semiconductor and the metal [84,85,86].

**Figure 11:**
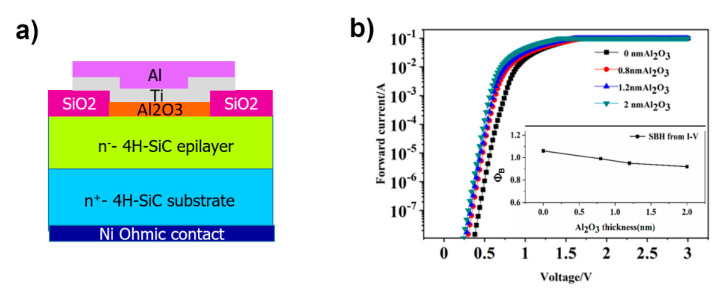
(**a**) Schematic view of a Ti/4H-Si Schottky diode with the insertion of an ultrathin Al_2_O_3_ layer between metal and semiconductor surface. (**b**) Forward I–V characteristics of the Ti/4H-SiC Schottky contact with increasing thickness of the inserted Al_2_O_3_-layer (0, 0.8, 1.2 and 2 nm). The trend of the Schottky barrier height as function of Al_2_O_3_ thickness is reported in the inset. Panel (**b**) is adapted with permission from Ref. [86]. Copyright 2021 Elsevier Ltd.

For example, Shi et al. [86] demonstrated that the presence of an ultrathin Al_2_O_3_ layer, deposited on the semiconductor surface by atomic-layer deposition before the metal stack (Al 300 nm/Ti 100 nm) annealed at in Ar at 300 °C for 5 min (Figure 11a), enabled a reduction of the barrier height. Three different oxide-layer thicknesses were investigated in that work (0.8, 1.2 and 2 nm). The forward I–V characteristics, shown in Figure 11b for all the tested Al_2_O_3_ thicknesses, indicated a reduction of the Schottky barrier height with the increase of the Al_2_O_3_ thickness, down to a value lower than 1 eV (inset of Figure 11b). In particular, the cross-section TEM analyses showed that the insertion of Al_2_O_3_ reduces the diffusion of Ti into 4H-SiC and, hence, the possible occurrence of solid-state reactions between metal and semiconductor. In this way, the formation of new titanium silicide and carbide phases is prevented, thus resulting in an improvement of the interface homogeneity.

**Figure 12:**
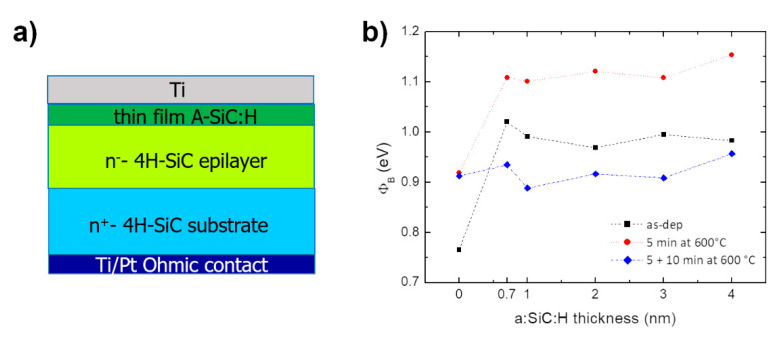
(**a**) Schematic view of a Ti/4H-SiC contact with an ultrathin amorphous SiC:H layer inserted between Ti and 4H-SiC. (**b**) Barrier height *ϕ_B_* for different thicknesses of the amorphous layer and duration of the annealing treatment. Panel (**b**) adapted with permission from Ref. [87]). Copyright 2021 Elsevier Ltd.

In another case, the insertion of an ultrathin amorphous-hydrogenated SiC layer (a-SiC:H) in the Ti/4H-SiC contact has been assessed with promising results (see schematic view in Figure 12a) [87]. The amorphous layer, with a thickness between 0.7 and 4 nm, was grown on the 4H-SiC surface by means of plasma-enhanced chemical vapor deposition prior to Ti deposition. Thermal annealing in a vacuum at 600 °C was also performed. The value of the Schottky barrier height varied between 0.78 and 1.16 V. These values, derived from room temperature I–V measurements, are reported in Figure 12b. As one can see, the Schottky barrier height depends on the amorphous layer thickness and thermal annealing duration. Specifically, while a slight influence of the amorphous layer thickness was observed on the Schottky barrier value, the duration of the 600 °C annealing, supposed to result in the formation of the Ti_5_Si_3_ phase [88], had a more significant impact on the barrier height. In particular, the lowest barrier value was obtained after the longest annealing treatment.

**Figure 13:**
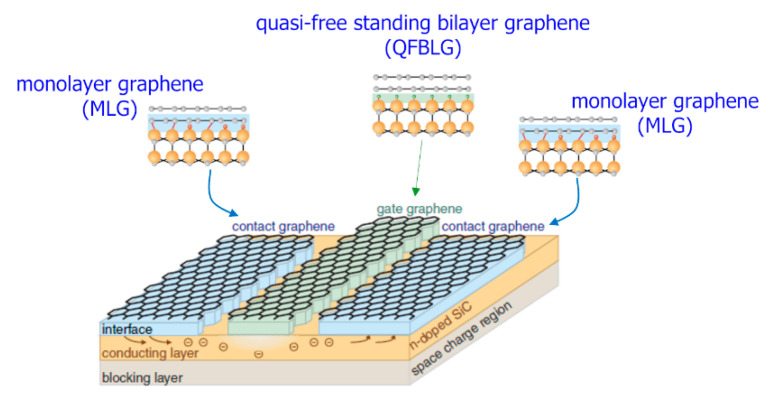
Schematic view of graphene/4H-SiC-based transistor, with two different interfaces, i.e., MLG and QFBLG contact acting as ohmic and Schottky gate contacts, respectively. Figure adapted with permission from Ref. [94]). Copyright 2012 Nature Portfolio.

Another method to modify, at atomic level, the surface where the Schottky contact is formed, consisted of the graphitization of the 4H-SiC surface [89,90]. It is known that as-grown monolayer graphene (MLG) on hexagonal SiC consists of a buffer layer (BL), similar to graphene but still covalently bond to SiC, plus a graphene overlayer [91]. The as-grown MLG contact exhibits ohmic characteristics, which have been explained by a low Schottky barrier height (*ϕ_B_* = 0.36 ± 0.1 eV) with SiC [92]. Such a low *ϕ_B_* value has been ascribed to the positively charged dangling Si bonds at the BL/SiC interface, which cause a Fermi level pinning of graphene close to the SiC conduction band, as well as a high n-type doping of graphene itself (*n* ≈ 1 × 10^13^ cm^−2^) [92]. Differently, after an annealing treatment of the MLG in H_2_-atmosphere, the covalent bonds between the BL and SiC break up and H_2_ saturates the dangling bonds, converting the electrically inactive BL into an additional real graphene layer [93]. This quasi-freestanding bilayer graphene (QFBLG) is moderately hole-doped (*p* ≈ 8 × 10^12^ cm^−2^) and provides a Schottky contact to 4H-SiC [94]. As discussed by Hertel et al. [94], these two different graphene/4H-SiC interfaces can be used side-by-side on the same chip in a real 4H-SiC-based MESFET device, as illustrated in Figure 13. Specifically, the MLG on 4H-SiC is used as ohmic contact for the source and drain electrodes (marked as “contact graphene”), while the QFBLG/4H-SiC–Schottky interface serves as a gate electrode (marked as “gate graphene”) [94].

In this system, nanoscale conductive atomic force microscopy (C-AFM) on QFBLG showed a dependence of the Schottky barrier height on the diode area, from values in the range (0.9–1) eV obtained for large contacts, up to values approaching ~1.5 eV for the smallest contacts. The behavior of this kind of contact was explained by considering that SiC step edges and facets are preferential current paths causing the effective lowering of the barrier. The reduced barrier height in these regions can be explained in terms of a reduced doping of QFBLG from SiC substrate at (11–20) step edges with respect to the p-type doping on the (0001) terraces [93].

**Figure 14:**
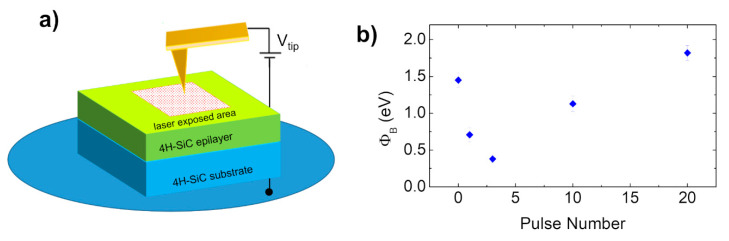
Nanoscale current–voltage characterization (by PeakForce TUNA mode of AFM) of Au/4H-SiC contact to laser-irradiated semiconductor surface: (**a**) scheme of the nanoscale current–voltage measurement set-up; (**b**) Schottky barrier height values extrapolated by I–V analysis for 4H-SiC surface irradiated with different pulse numbers. Figure adapted with permission from Ref. [95]. Copyright 2021 Elsevier Ltd.

A final example regards the work of Lin et al. [95], who explored a new way to fabricate tunable Schottky diodes with ns-pulsed excimer laser (193 nm)-modified n-type single-crystal 4H-SiC. The diodes were analyzed both by macroscopic I–V measurements by using Au-layer as electrode and by nanoscale characterization by means of atomic force microscopy in PeakForce TUNA configuration, this latter schematized in Figure 14a. Particularly, as noticed from the macroscopic I–V characterization on pristine and irradiated contacts, the most notable change in the I–V behavior was observed for the contact exposed to 2 J/cm^2^ (not shown here). For the contact irradiated at such fluence, the nanoscale I–V characterization for different numbers of pulses (1–20 pulses), directly on the bare laser-exposed surface of the semiconductor, showed a rectifying electrical behavior of the contact, with the Schottky barrier increasing from 0.38 up to 1.82 V in the range 3–20 pulses (reported in Figure 14b). A combined analysis with Raman spectroscopy for the sample irradiated at 2 J/cm^2^ demonstrated a graphitization of the 4H-SiC surface after laser irradiation, which is probably at the base of the barrier height increase in contact to the laser-modified 4H-SiC surface. For fluence as high as 5 J/cm^2^, the appearance of the peak corresponding to monocrystalline silicon (~520 cm^−1^) was observed.

### 3.2. N-Type Doping of the Interface

The capability of 4H-SiC to sustain a high electric field (if compared to conventional semiconductors, such as Si) enables the possibility of tailoring the Schottky barrier height by varying the doping concentration (and hence the electric field) below the contact. If, under reverse bias, the effect of a larger electric field has been widely investigated with an experimentally observed larger leakage current explained by the *TFE* model [96,97] and mitigated by the use of the JBS layout, under forward bias, the effect of a modification of the electric field requires deeper understanding. For instance, ion-irradiation-induced damage below the interface in Ti/4H-SiC Schottky diodes showed the possibility of increasing the barrier height by a deactivation of the dopant and a reduction of the electric field at the interface following a re-ordering of the crystal structure [98].

On the other hand, as mentioned above, a way to increase the electric field consists of increasing the doping concentration in the semiconductor below the contact layer. In this context, it is interesting to study the effects on the barrier height and carrier transport mechanisms in a heavily-doped 4H-SiC layer [19,99]. Hara et al. [19] studied the dependence of the barrier height and forward carrier transport mechanism on the doping concentration *N_D_* in Ni/SiC Schottky barrier diodes with 4H-SiC epitaxial layer. In particular, they investigated a range of doping concentrations, varying from 6.8 × 10^15^ up to 1.8 × 10^19^ cm^−3^. The increase in the doping concentration entailed a shift of the forward I–V characteristics (that means larger current observed for higher doping concentration for a given voltage value). This shift corresponded to a lower turn-on voltage, increasingly stronger from the lightly-doped sample (*N_D_* = 6.8 × 10^15^ cm^−3^) to the heavily-doped sample (*N_D_* = 1.8 × 10^19^ cm^−3^). On the other hand, a modification was observed for the predominant current transport mechanism, sweeping from TE to a *TFE* mechanism for a higher doping concentration (*N_D_* > 2.6 × 10^17^ cm^−3^).

**Figure 15:**
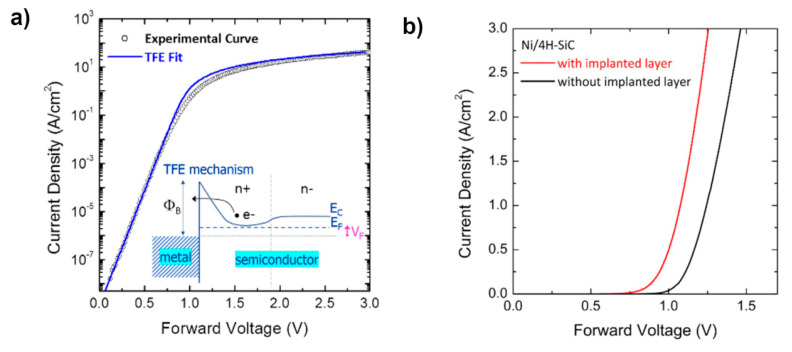
(**a**) Forward current density–voltage characteristics (open symbols) for Ni/n-type implanted-4H-SiC Schottky diode and fitting curve according to the *TFE* model (continuous line). In the inset, schematic energy band diagram for the metal/4H-SiC contact under forward bias, according to the *TFE* current transport mechanism. (**b**) Forward current density–voltage characteristics of the Ni Schottky contacts to n-type 4H-SiCwith or without a heavily doped n-type implanted layer. (Figure extracted from Ref. [100]).

The predominance of the *TFE* mechanism for Schottky contacts on a heavily-doped 4H-SiC layer was also demonstrated for Ni/4H-SiC with a n+-type implanted layer of 4H-SiC (*N_D_* = 1.97 × 10^19^ cm^−3^) [100], whose forward *J*-*V_F_* characteristics are shown in Figure 15a. The inset reports the schematic energy band diagram for the metal/4H-SiC interface when a *TFE* current transport mechanism is predominant. This contact exhibited a lower value of turn-on voltage if compared to a reference Ni/4H-SiC contact formed on the 4H-SiC epilayer without implanted layer and standard epitaxial layer doping concentration, as clearly highlighted by the graph in Figure 15b. The possible increase of the leakage current could be mitigated by an appropriate choice of the device layout, as in the JBS diode. This last point was theoretically investigated in Ref. [100].

## 4. Conclusions

In this paper, we overviewed some approaches applied in the 4H-SiC Schottky contact development in order to improve the performance of the Schottky devices.

After a short discussion on the fundamentals of the metal/4H-SiC Schottky contact formation and the typical electrical characterization by I–V measurements, we pointed out the well-established technology of Schottky diodes, using Ti or Ni-based Schottky barriers and discussed the current solutions, including the most promising low work function and highly chemically stable metallization schemes and appropriate diode layouts. Then, we presented some unconventional methods based on the manipulation of the metal/semiconductor interface and aimed at an improved control of the Schottky properties of the contact. As a matter of fact, although the metal/4H-SiC system has been studied for a long time, many aspects in the contact formation are still unclear and require a deeper understanding, both from a fundamental and a technological standpoint, in order to obtain superior control of the Schottky contact electrical properties.

Nevertheless, some solutions have shown interesting outcomes. For instance, the introduction of metal nanoparticles in the metal layer has been considered for the advantages given in terms of barrier reduction. Other solutions act on the semiconductor side, for example, with treatments before metal deposition, in order to homogenize the surface and narrow the barrier heights and ideality factor distribution. The effects on the Schottky barrier related to an increase of the doping density of the semiconductor layer have also been investigated. Although these are early studies, they are very promising for the practical implications in Schottky diode technology. In fact, in addition to an improvement of the electrical properties in terms of uniformity, these solutions addressed the superior control of the Schottky barrier height, with the ultimate capability to tailor and tune its value. Besides the possibility of obtaining insight into the physical characteristics of the Schottky contact, this aspect is of particular interest for the device makers, for the development of a new class of Schottky diodes with tailored characteristics.

## Figures and Tables

**Table 1:** Schottky barrier height for metal/n-type 4H-SiC system for different metals. The values were determined by I–V measurements on Schottky diodes.

Metal	*ϕ_B_* (eV)	Thermal Treatment	Reference
Ta	1.10	none	[34]
Ti	0.95	none	[35,36]
Ti	0.78	none	[37]
Ti	0.96	none	[38]
Ti	1.15	600 °C for 10 min in Ar	[37]
Ti	0.95	500 °C for 60 h in vacuum	[35]
W	1.11	none	[39]
W	1.14–1.25	475–700 °C for 10 min in N_2_	[39]
W	1.17	none	[37]
W	1.09	600 °C for 10 min in Ar	[37]
W	1.11	500 °C in N2	[40]
Mo	1.04	none	[29]
Mo	1.11	none	[37]
Mo	1.21	600 °C for 10 min in Ar	[37]
Mo	1.17	none	[41]
Mo	1.01	400 °C for 30 min in Ar	[41]
Ni	1.45	none	[29]
Ni	1.62	none	[27]
Ni	1.52	none	[38]
Ni	1.52	400 °C, RTA	[29]
Au	1.73	none	[27]
Ir	1.31	none	[42]
Pt	1.39	none	[43]
Pt	1.72	200 °C	[44]

## Data Availability

The data that support the findings of this study are available from the corresponding author upon reasonable request.
